# Annual of the Universal Medical Sciences

**Published:** 1896-02

**Authors:** 


### Book Notices.

ANNUAL OF THE UNIVERSAL MEDICAL SCIENCES. A Yearly Report of the Progress of the General Sanitary Sciences Throughout the World. Edited by Charles E. Sajous, M. D., and seventy associate editors, assisted by over two hundred corresponding editors, collaborators, and correspondents. Illustrated with chromo-lithographs and maps. In five volumes of more than six hundred pages each. Price per set, cloth, $15.00. The F. A. Davis Co., Publishers, 1914-1916 Cherry St., Philadelphia. 1895.

This is the eighth issue of the Annual, and although it is late in coming from the press, it will be received with gladness by its thousands of subscribers. This issue seems to come fully up to the high standard established by its predecessors, and we can find no fault with the work of the editor, his associate editors, corresponding editors, collaborators, or publishers. The work of examining all of the medical journals, monographs, books, etc., that have appeared during the past year, and selecting from them that which is new and valuable, thus making a yearly report of the progress of medical science in all of its branches throughout the world, is a task of enormous proportions. Such a task is possible only when a large number of men from all parts of the world divide this work, each one taking a particular part of it, yet all working together to make it full and complete. The Annual is the result of such a company of competent and trained men, all working together, and in it we have the recent valuable additions that have been made to medical science. The five volumes cover the whole domain of medicine and surgery, the contents of the series reading as follows:

Vol. I.—Diseases of the Lungs and Pleura, by Dr. J. C. Wilson and Dr. A. A. Eshner; Diseases of the Heart and Blood Vessels, by Dr. H. F. Vickery; Diseases of the Mouth, Stomach, Liver and Pancreas, by Dr. A. Rubino; Cholera; Diseases of the Intestines and Peritoneum, by Drs. J. P. C. Griffith and A. Hand, Jr.; Animal Parasites and their Effects, by Dr. C. S. Dolley; Diseases of the Kidneys, Bladder and Adrenals; Urinalysis, by Dr. M. Lannois; Diabetes Mellitus, by Dr. R. Lépine. Fevers, by Dr. F. Semeleder; Diphtheria, Croup, Pertusis and Parotitis, by Dr. J. L. Smith and Dr. F. M. Warner; Scarlet Fever, Measles, Varicella and Rötheln, by Dr. C. S. Witherstine; Rheumatism and Gout, by Dr. N. S. Davis; Diseases of the Blood and Spleen, by Drs. F. P. Henry and A. Stengel.

Vol. II.—Diseases of the Brain, by Drs. L. C. Gray, W. B. Pritchard and R. C. Schultz; Diseases of the Spinal Cord, by Dr. H. Obersteiner; Peripheral Nervous Diseases, Muscular Dystrophies, and General Neuroses, by Dr. P. Sollier; Traumatic Neuroses, by Dr. A. J. Booth; Mental Diseases, by Dr. George H. Rohé; Inebriety, Morphinism and kindred Disorders, by Dr. Norman Kerr; Diseases of the Uterus, Tubes, Ovaries and Pelvic Tissues, by Dr. E. E. Montgomery; Diseases of the Vagina and External Genitals, by Drs. J. M. Baldy and W. A. N. Dorland; Diseases of Pregnancy, by Dr. A. Lutaud; Obstetrics and Puerperal Diseases, by Drs. P. Budin and L. Merle; Diseases of the Newborn; Teratology, by Dr. A. F. Currier; Dietetics of Infancy and Childhood; Infantile Disorders, by Dr. W. A. Edwards.

Vol. III.—Surgery of the Brain, Spinal Cord and Nerves, by Drs. L. S. Pilcher and Samuel Lloyd; Thoracic Surgery, by Drs. J. McF. Gaston and J. McF. Gaston, Jr.; Surgery of the Abdomen, by Drs. Wm. T. Bull and Wm. B. Caley; Diseases of the Rectum and Anus, by Dr. C. B. Kelsey; Surgical Diseases of the Genito-Urinary Apparatus in the Male, by Drs. E. L. Keyes and E. Fuller; Syphilis, by Drs. J. Wm. White, Wm. H. Furness and H. M. Hiller; Orthopædic Surgery, by Drs. L. A. Sayre and R. H. Sayre; Amputations, Resections and Plastic Surgery; Diseases of the Bones and Joints, by Drs. P. S. Connor and L. Freeman; Fractures and Dislocations, by Dr. L. A. Stimson; Diseases and Injuries of Arteries and Veins, by Dr. C. Fenger; Oral Surgery, by Dr. R. Matas; Tumors and Surgical Mycoses, by Dr. E. Laplace; Surgical Diseases, by Drs. L. McL. Tiffany and R. B. Warfield; Surgical Dressings and Antiseptics, by Dr. F. Van Imschoot; Anæsthetics, by Dr. D. W. Buxton.

Vol. IV.—Diseases of the Skin, by Dr. A. Van Harlingen; Diseases of the Eye, by Drs. C. A. Olivier and Wm. C. Posey; Diseases of the Ear, by Drs. C. S. Turnbull and A. A. Bliss; Diseases of the Nasal and Accessory Cavities, Pharynx, Larynx Trachea, and Œsophagus, by Dr. C. E. Sajous; Intubation of the Larynx, by Dr. James O'Dwyer; Diseases of the Thyroid Gland, by Dr. J. Payson Clark; Legal Medicine and Toxicology, by Dr. F. Winthrop Draper; Medical Demography, by Dr. F. Levison; Bacteriology, by Dr. Harold C. Ernst.

Vol. V.—General Therapeutic and Pharmaceutical Chemistry, by Dr. G. Dujardin-Beaumetz and Dr. H. Dubief; Experimental Therapeutics, by Drs. H. A. Hare and David Cerna; Electro-Therapeutics, by Dr. A. D. Rockwell and Dr. James E. Nichols; Gynæcological Electro-Therapeutics, by Drs. G. Apostoli and Jules Grand; Hydrotherapy, Climatology and Balneology, Drs. Simon Baruch and Frank H. Daniels; Hygiene and Epidemiology, by Surgeon-General Walter Wyman and Dr. Charles E. Banks; Anatomy, by Dr. L. Testut and Dr. E. Vialleton; Normal Histology and Microscopical Technology, by Dr. Charles E. Sajous; Physiology, by Drs. W. H. Howell and G. P. Dreyer. H.

